# Shiga toxin 2-induced intestinal pathology in infant rabbits is A-subunit dependent and responsive to the tyrosine kinase and potential ZAK inhibitor imatinib

**DOI:** 10.3389/fcimb.2012.00135

**Published:** 2012-11-08

**Authors:** Samuel M. Stone, Cheleste M. Thorpe, Amrita Ahluwalia, Arlin B. Rogers, Fumiko Obata, Aimee Vozenilek, Glynis L. Kolling, Anne V. Kane, Bruce E. Magun, Dakshina M. Jandhyala

**Affiliations:** ^1^Division of Geographic Medicine and Infectious Diseases, Tufts Medical Center, and Tufts University School of MedicineBoston, MA, USA; ^2^VA Long Beach Healthcare SystemLong Beach, CA, USA; ^3^Department of Pathology and Laboratory Medicine, The University of North Carolina School of MedicineChapel Hill, NC, USA; ^4^Department of Microbiology and Immunology, University of Maryland School of MedicineBaltimore, MD, USA; ^5^Department of Internal Medicine/Nephrology, University of VirginiaCharlottesville, VA, USA; ^6^Phoenix Laboratory, Tufts Medical CenterBoston, MA, USA; ^7^Department of Cell and Developmental Biology, Oregon Health and Science UniversityPortland, OR, USA

**Keywords:** Shiga toxin, globotriaosylceramide, inflammation, colon, rabbit, p38, imatinib

## Abstract

Shiga toxin producing *Escherichia coli* (STEC) are a major cause of food-borne illness worldwide. However, a consensus regarding the role Shiga toxins play in the onset of diarrhea and hemorrhagic colitis (HC) is lacking. One of the obstacles to understanding the role of Shiga toxins to STEC-mediated intestinal pathology is a deficit in small animal models that perfectly mimic human disease. Infant rabbits have been previously used to study STEC and/or Shiga toxin-mediated intestinal inflammation and diarrhea. We demonstrate using infant rabbits that Shiga toxin-mediated intestinal damage requires A-subunit activity, and like the human colon, that of the infant rabbit expresses the Shiga toxin receptor Gb_3_. We also demonstrate that Shiga toxin treatment of the infant rabbit results in apoptosis and activation of p38 within colonic tissues. Finally we demonstrate that the infant rabbit model may be used to test candidate therapeutics against Shiga toxin-mediated intestinal damage. While the p38 inhibitor SB203580 and the ZAK inhibitor DHP-2 were ineffective at preventing Shiga toxin-mediated damage to the colon, pretreatment of infant rabbits with the drug imatinib resulted in a decrease of Shiga toxin-mediated heterophil infiltration of the colon. Therefore, we propose that this model may be useful in elucidating mechanisms by which Shiga toxins could contribute to intestinal damage in the human.

## Introduction

Shiga toxin-producing *Escherichia coli* (STEC) are a heterogenous group of *E. coli* strains responsible for food- and water-borne illness worldwide. Depending on the strain, approximately 5–22% of infected individuals will suffer severe illnesses that can result in permanent disability or death (Paton et al., 1998; Kulasekara et al., 2009; Frank et al., 2011). Severe illnesses attributed to STEC-associated sequelae include hemorrhagic colitis (HC) and the hemolytic uremic syndrome (HUS), the predominant cause of renal failure in US children (Siegler, 2003). Other than volume expansion during the diarrheal phase, no approved specific preventative treatments exist for STEC-associated HUS.

Shiga toxins (Stxs) are the key virulence factors responsible for promoting severe disease during STEC infection. Stxs are AB5 toxins consisting of a single A-subunit non-covalently bound to 5 B-subunits. The B-subunits are necessary for binding of the toxin to the surface of the host cells via interaction with neutral glycolipids with the glycosphingolipid receptor globotriaosylceramide (Gb_3_) being the major receptor (Lingwood et al., 2010). Once bound, the toxin undergoes receptor-mediated endocytosis and is transported retrograde through the early endosome, the Golgi apparatus, and to the endoplasmic reticulum (ER). Somewhere between the early endosome and the trans-Golgi network, the enzymatically active portion of the A-subunit is proteolytically cleaved possibly by furin into an A1 fragment which remains bound to the A2 fragment and non-covalently associated B-subunits via an intramolecular disulfide bond (Garred et al., 1995a,b; Tam and Lingwood, 2007). Eventually the disulfide bond is reduced, possibly in the ER (Spooner and Lord, 2012), and the enzymatically active A1 fragment is translocated to the cytoplasm, where its *N*-glycosidase activity results in the depurination of a single adenine residue located in the alpha-sarcin/ricin loop of the 28S ribosomal RNA (Endo and Tsurugi, 1987; Endo et al., 1987, 1988). This depurination event results in the cessation of protein synthesis in the translational elongation phase and activates a proinflammatory signaling cascade referred to as the ribotoxic stress response (RSR) (Iordanov et al., 1997).

The RSR is defined as the activation of MAPKinases by certain protein synthesis inhibitors including Shiga toxins, ricin, anisomycin, doxorubicin, and the trichothecene mycotoxins (Iordanov et al., 1997, 1998; Shifrin and Anderson, 1999; Smith et al., 2003; Zhou et al., 2003; Sauter et al., 2010). The RSR has been shown to result in a paradoxical up-regulation of several cytokines despite a decrease in global protein synthesis (Thorpe et al., 1999b, 2001; Foster and Tesh, 2002; Cherla et al., 2006; Gonzalez et al., 2006). Finally, activation of pro-apoptotic signaling has also been shown to occur following the activation of the RSR (Smith et al., 2003). However, the role of the RSR in Shiga toxin-mediated pathology *in vivo* has not been determined.

As STEC strains are generally non-invasive, it is believed that HUS results from the systemic uptake of Shiga toxins and possibly other virulence factors (e.g., LPS) from the intestinal lumen. Both transcellular and a paracellular route have been noted as pathways by which Stx may enter the systemic circulation from the intestinal lumen (Acheson et al., 1996; Hurley et al., 2001; Malyukova et al., 2009). Data suggests that Stx can enter and cross the intestinal epithelium via receptor independent macropinocytosis, (Malyukova et al., 2009; Lukyanenko et al., 2011). This transcellular transcytosis may represent the major pathway, at least during the early stages of infection, by which Stx enters the systemic circulation. Alternatively, Stx and/or other STEC virulence factors may contribute to Stx systemic uptake by increasing the overall state of intestinal inflammation. It has been demonstrated *in vitro* that a decrease in epithelial barrier function to Stx correlates with neutrophil transmigration across polarized intestinal epithelial cells (Hurley et al., 2001), suggesting that Stxs could cross the intestinal epithelium via a paracellular route that is promoted by inflammation. Therefore, the inflammation and damage to the intestine that occurs during HC (Griffin et al., 1990) may compromise intestinal barrier function and promote systemic disease (i.e., HUS). However, the exact mechanism(s) by which Shiga toxins themselves contribute to this compromise of gut barrier function remains unclear.

In order to intoxicate and thereby induce an inflammatory response, Stx must bind and enter cells via receptor-mediated endocytotic pathways (Jacewicz et al., 1994; Khine et al., 2004; Zumbrun et al., 2010). Gb3 is the best characterized cell surface receptor through which Stx binds and enters cells (Waddell et al., 1988, 1990; Sandvig et al., 1991; Lingwood et al., 1998). While it has been shown in rabbits that Gb_3_ is maturationally regulated in the small bowel (Mobassaleh et al., 1994), nothing is known concerning Gb_3_ levels in the rabbit colon. In the present study, we use an infant rabbit oral intoxication model (Ritchie et al., 2003) with purified Stx2 and an enzymatically inactive Stx2A_Y177S,E167Q_B to investigate the role of Stx2 subunits in toxin-induced colonic inflammation. We assess Gb_3_ expression in the infant rabbit colon and demonstrated Stx2-mediated activation of the MAPK p38 in intestinal tissue. Finally, we investigate whether treatment with inhibitors of the RSR may prevent or lessen Stx2-induced intestinal inflammation.

## Materials and methods

### Antibodies, inhibitors, and other reagents

Antibodies to phospho-p38 (cat# sc-7973) were purchased from Santa Cruz Biotechnology Inc. (Santa Cruz, CA). The DNA plasmid expressing Stx2A_Y177S,E167Q_B (pNR100) was provided to us by Dr. Tom Obrig of the University of Maryland (Baltimore, MD) and its construction was previously described by Wen et al. (2006). Both Stx2 and Stx2A_Y77S,E167Q_B were purified by Dr. Anne Kane of the Phoenix Lab at Tufts Medical Center (Boston, MA) using previously described techniques (Donohue-Rolfe et al., 1989). The ZAK specific inhibitor 7-[3-fluoro-4-aminophenyl-(4-(2-pyridin-2-yl-5, 6-dihydro-4Hpyrrolo[1,2-b]pyrazol-3-yl))]-quinoline DHP-2 was synthesized by Albany Molecular Research (Albany, NY). Imatinib mesylate (Novartis, Cambridge, MA) was purchased from the Tufts Medical Center Pharmacy. The p38 inhibitor SB203580 was purchased from LC Laboratories (Woburn, MA).

### Cell culture

Tissue culture media and reagents were obtained from Gibco BRL-Life Technologies (Grand Island, NY). HCT-8 cells were maintained at 37°C in an atmosphere of 5% CO_2_ in RPMI 1640 medium supplemented with 10 mM HEPES, 1 mM sodium pyruvate, 10% heat-inactivated FCS, 50 IU/ml of penicillin, and 50 μg/ml of streptomycin and used at 90–100% confluence.

### Animal protocols

All procedures involving live animals were pre-approved by our institutional animal care and use committee. Two-day old New Zealand White rabbits were obtained with doe from Millbrook Labs (Amherst, MA). Each litter was housed as a group and nursed by the mother. Three day old rabbits were given Stx2, Stx2A_Y77S, E167Q_B, or heat-inactivated Stx2 intragastrically in doses of 1.0 mg/kg of rabbit weight daily for 2 consecutive days, followed by euthanasia and necropsy. The first day of toxin administration was designated “day 0”. For studies involving inhibitors, rabbits were given either vehicle, 20 mg/kg SB203580 every 12 h by intraperitoneal injection, 30 mg/kg DHP-2 every 24 h by oral gavage, or 1.5 mg/kg imatinib every 12 h by oral gavage, beginning on day 2 of life (day –1), then re-dosed every 12 h (vehicle, SB203580 or imatinib) or every 24 h (vehicle or DHP-2), until euthanasia and necropsy. Infant rabbits were weighed daily and monitored twice daily for signs of illness. Diarrhea was scored as follows: none = no diarrhea (normal pellets are dark green, hard, and formed); mild = diarrhea consisting of light heel staining; or moderate = diarrhea consisting of staining of the perineum, hind legs, and heels with occasional unformed stool adherent. All rabbits were euthanized by isoflurane inhalation until unresponsive to painful stimulus, followed by intracardiac injection of euthasol (Virbac Animal Health Inc., Fort Worth, TX).

At necropsy, the intestinal tract from the anus to the mid-colon was removed, and samples were obtained for lipid extraction, histology, and/or immunohistochemistry. To eliminate any litter-specific effects, all experimental conditions (treatments and controls) were represented, in each litter treated. Thus small litters, large litters (>10 pups), or litters in which there were extreme variations in pup size were not used. In order to achieve sufficient power, 5 different litters consisting of 32 animals were used for the study comparing Stx2 with the enzymatically inactive Stx2A_Y177S,E167Q_B, 4 litters consisting of 31 animals were used for the SB203580 study, 3 litters consisting of 20 animals were used for the DHP-2 study, and 8 litters consisting of 49 animals were used for the imatinib study.

### Histology

Except for the tissue used for Gb_3_ analysis (detailed further below), all other distal colon tissue obtained at necropsy was immediately fixed in 10% buffered formalin, then paraffin-embedded. Sections 5 μm thick were obtained and mounted on glass slides. Slides for histological analysis of inflammation were stained with hematoxylin and eosin (H&E). The slides were then blindly scored by a veterinary pathologist on four criteria including heterophil, epithelial defects, edema, and congestion/hemorrhage, or on 5 criteria that included heterophils, epithelial defects, congestion/edema/hemorrhage, mononuclear cells, and atrophy. A scale from 0 to 4 was used with 0 being the least severe or lowest, and 4 being the most severe or highest.

### Lipid extraction and thin layer chromatography

Tissues from the large intestine (distal and mid-colon), cecum, and ileum were collected and processed to isolate neutral lipids according to previously described methods (Nutikka et al., 2003; Kolling et al., 2008). Saponified neutral lipid fractions were separated by thin layer chromatography and Gb_3_ detected using the toxin overlay method (Nutikka et al., 2003). Lipid standards were purchased from Matreya LLC (Pleasant Gap, PA). Toxin overlays were conducted using Stx1B and an anti-polyclonal Stx1B antibody (gift from Dr. C. A. Lingwood, Hospital for Sick Children, Toronto, ON).

### Immunohistochemistry for Gb_3_

Anti-Gb_3_ immunohistochemistry was performed on paraffin embedded sections of rabbit colon using the method of Kolling with modifications (Kolling et al., 2008). Briefly, tissues were fixed with 4% paraformaldehyde (PFA) in phosphate buffered saline (PBS) for 24 h at room temperature and washed with PBS. Tissues were dehydrated using an acetone gradient; 25% for 10 min × 2, 75% for 10 min × 2, 95% for 20 min × 2, and 100% for 20 min × 2 and then immersed in xylenes for 5 min × 2 prior to paraffin embedding. The paraffinized blocks were sectioned to 3 μm. Following antigen retrieval using a microwave oven, the Vectastain ELITE ABC (VECTOR laboratories, Burlingame, CA) reagent protocol was followed with the exception that endogenous IgM was blocked with goat anti-rat IgM (H&L)F(ab′)_2_ using a 1:50 dilution (American Qualex, San Clemente, CA). The primary antibodies used were CD77/Gb_3_ monoclonal rat IgM antibody (clone 38.13, Immunotech/Beckman Coulter, Brea, CA) and as a control, an isotype matched rat IgM from Chemicon/Millipore (Billerica, MA). The secondary antibody used was anti-rat IgM-biotin conjugate from American Qualex.

### Immunohistochemistry for phospho-p38

Immunostaining was performed using formalin-fixed paraffin-embedded 5 μm thick intestinal sections. Sections were deparaffinized, hydrated, and subsequently subjected to microwave antigen retrieval using a target retrieval solution (Dako, Carpinteria, CA) at pH 10.00. Endogenous peroxidase was blocked with 3% H_2_O_2_/H_2_O, and a serum-free protein block (Dako) was used to prevent non-specific protein binding. Tissue sections were incubated overnight with antibodies against activated (i.e., phosphophorylated) p38 (1:100; Santa Cruz Biotech, CA) at 4°C, followed by biotinylated anti-mouse secondary antibody and a peroxidase-labeled streptavidin-biotin (Dako). The 3-amino-9-ethylcarbazole (AEC) substrate-chromogen (Dako) detection method for immunostaining was used and sections were counterstained with Mayer's hematoxylin. Specificity of staining was confirmed by omitting the primary antibody (negative controls).

### Terminal deoxynucleotidyltransferase biotin-dUTP nick and labeling (TUNEL) staining

To detect DNA fragmentation due to apoptosis, paraffin sections were stained using the ApopTagPlus Peroxidase *In Situ* apoptosis detection kit (Millipore, MA). In short, sections were deparaffinized and treated with proteinase K for antigen retrieval. After endogenous peroxidase blocking, digoxigenin (DIG)-conjugated nucleotides were incorporated to 3′-OH nicks of DNA using terminal deoxynucleotidyltransferase (TdT). The labeled DNA sites were visualized using peroxidase labeled anti-DIG and 3,3′-diaminobenzidine (DAB). DAB positive brown nuclei were counted and normalized to total viewing area.

### Statistical analysis

Histology scores are ordinal non-parametric data, and were analyzed either by One-Way ANOVA analysis for the data presented in Figure 2, or by the Mann–Whitney *U*-test (Figures 4 and 9) using Prism software (GraphPad, San Diego, CA).

## Results

### Heterophil infiltration and epithelial defects are dependent on Stx2 catalytic activity

Members of our group have previously reported that following oral administration of 100 μg/kg of Stx2, given as a repeated dose on day 0 and day 1, rabbits developed diarrhea by day 2 compared with animals dosed with heat-inactivated Stx2 (HI) as a control treatment (Ritchie et al., 2003). Furthermore, when compared to animals given HI, rabbits given purified Stx2 had significantly more heterophils in the distal colonic mucosa and more epithelial defects consisting of crypt abscesses, crypt ectasia, and distortion and intraluminal plugging of degenerating heterophils and epithelial cells.

**Figure 1:**
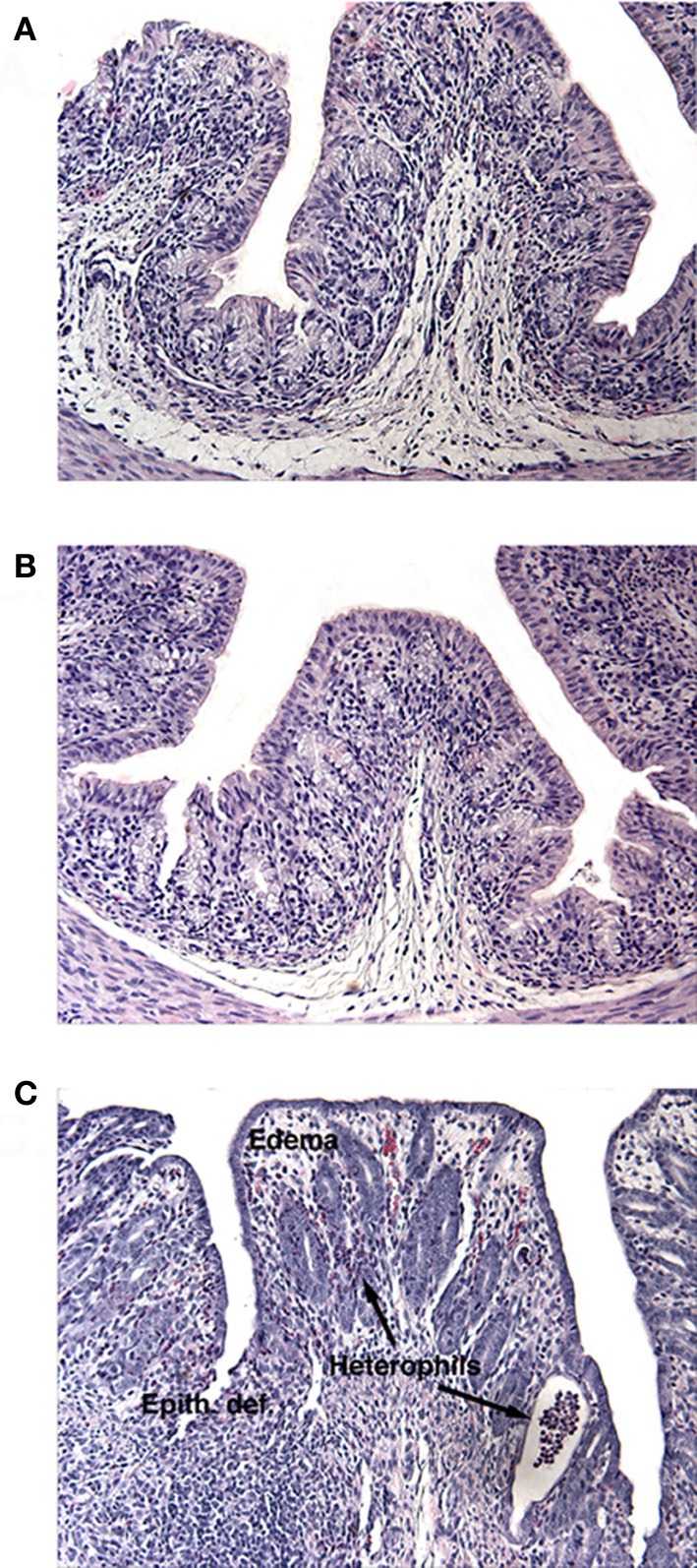
**H and E stained sections of infant rabbit colon following treatment with (A) HI, (B) Mut, or (C) Stx2.** Tissue from Stx2-treated rabbits show increased edema, epithelial defects (Epith def), and heterophil infiltration.

**Figure 2:**
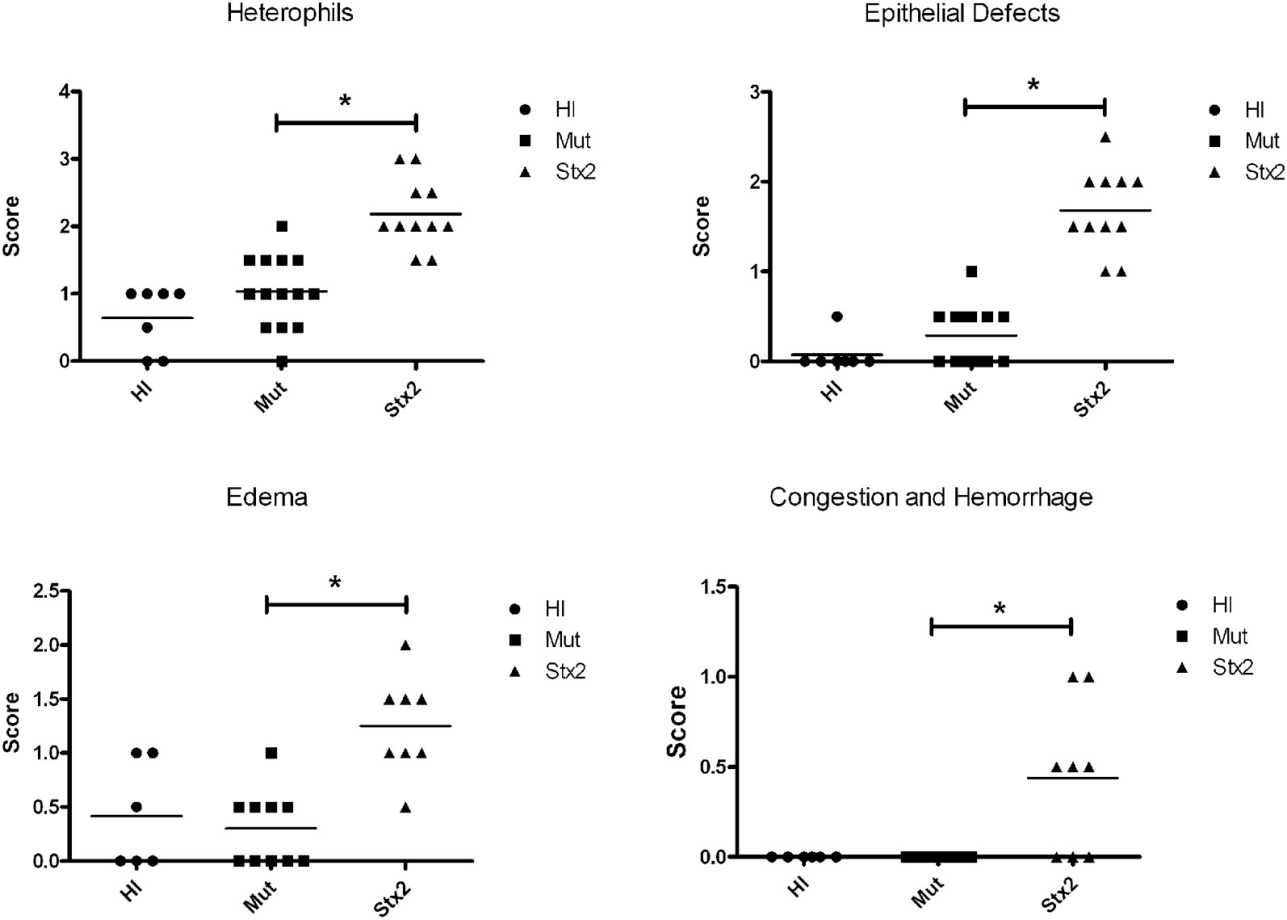
**Five litters composed of 32 animals were treated once a day for 2 days with 100 μg/kg of heat-inactivated Stx2 (HI) (*n* = 7), Stx2A_E167QY77S_B (Mut) (*n* = 14), or Stx2 (*n* = 11).** Blinded scoring was performed for heterophil infiltration and epithelial defects. Similarly, tissue was scored for edema and congestion/hemorrhage for tissue samples from four of the five litters (HI, *n* = 6; Mut, *n* = 10; Stx2 *n* = 8). ^*^Significance (*P*-value < 0.05) was determined by one-way ANOVA.

**Figure 3:**
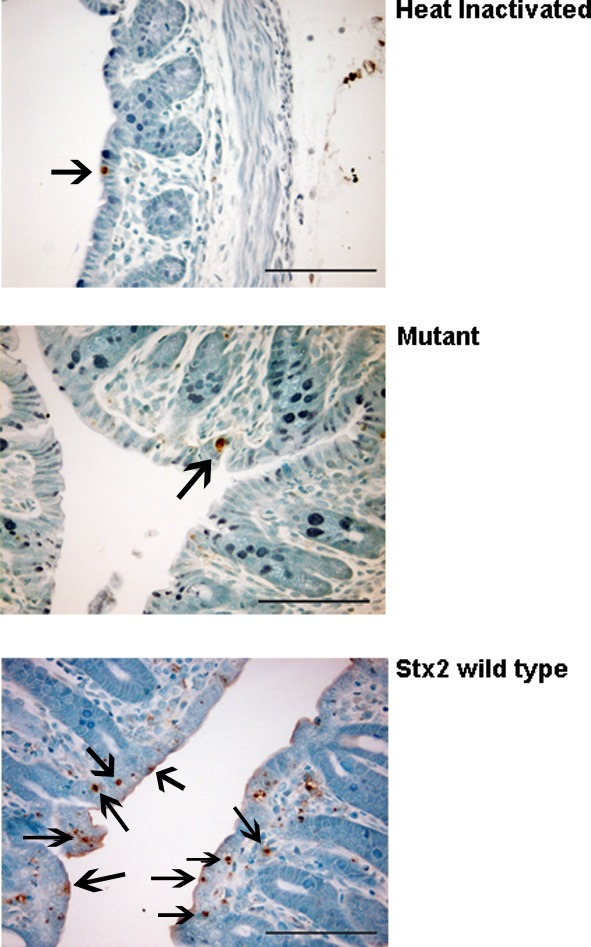
**Panels showing representative differences in TUNEL positive cells (arrows) in the colon of heat-inactivated Stx2-, mutant-, and wild type Stx2- treated animals**.

**Figure 4:**
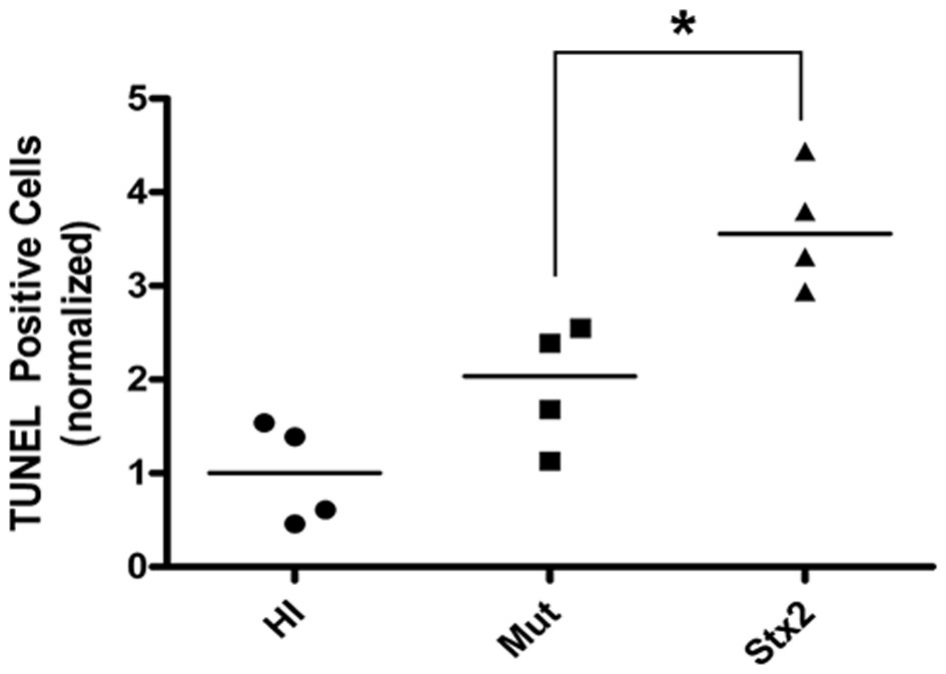
**Tissue sections from HI, Mut, and Stx2-treated animals (*n* = 4 for each from a single litter) were analyzed for apoptotic events by TUNEL staining.** For a given experiment, the number of apoptotic cells were counted per unit area and then normalized against those of the HI-treated animals. The normalized values are shown for each individual animal along with the median (horizontial line). ^*^Significance as determined by the Mann–Whitney *U*-test *P* = 0.029.

The results from several studies have suggested that the Stx B-subunit has the ability to promote innate immune signaling, apoptosis, and/or thrombotic microangiopathy (Mangeney et al., 1993; Nakagawa et al., 1999; Fischer et al., 2007; Huang et al., 2010). To determine if Stx2-induced intestinal damage was dependent on Stx2 A-subunit *N*-glycosidase activity, we compared the effects of oral Stx2 treatment with that of Stx2A_Y177S,E167Q_B (Mut), a Stx2 mutant toxin with active site mutations in the Stx2 A-subunit (Deresiewicz et al., 1992; Wen et al., 2006). In each of 5 litters of infant rabbits, individual animals were intoxicated once a day with 100 ug/kg of HI, Mut, or Stx2 for 2 days. Compared with rabbits intoxicated with Stx2 (Figures 1 and 2), there was a statistically significant (*P* < 0.05) decrease in heterophils, epithelial defects, edema, and congestion/hemorrhage observed in animals given Mut. These parameters were not significantly different (i.e., *P* > 0.05) between HI- vs. Mut-treated animals in any of the four categories. In agreement with these data, TUNEL staining of colon sections (Figures 3 and 4) from two of the 5 litters (*n* = 4 for each treatment) demonstrate that Stx2-treated animals had increased numbers of apoptotic cells compared with those treated with HI and Mut. In addition, most of the TUNEL positive cells in the Stx2-treated animals were found either apically near the brush border or between the brush border and basal lamina with relatively few being detected in the submucosa (data not shown). Since the B-subunit is present in both Stx2 and Mut preps, data suggests that in this model, Stx2-induced intestinal damage and apoptosis is largely dependent on A-subunit catalytic activity.

### Gb_3_ is detectable in the distal colonic epithelium in 5-day old infant rabbits and is affected by Stx2 treatment

**Figure 5:**
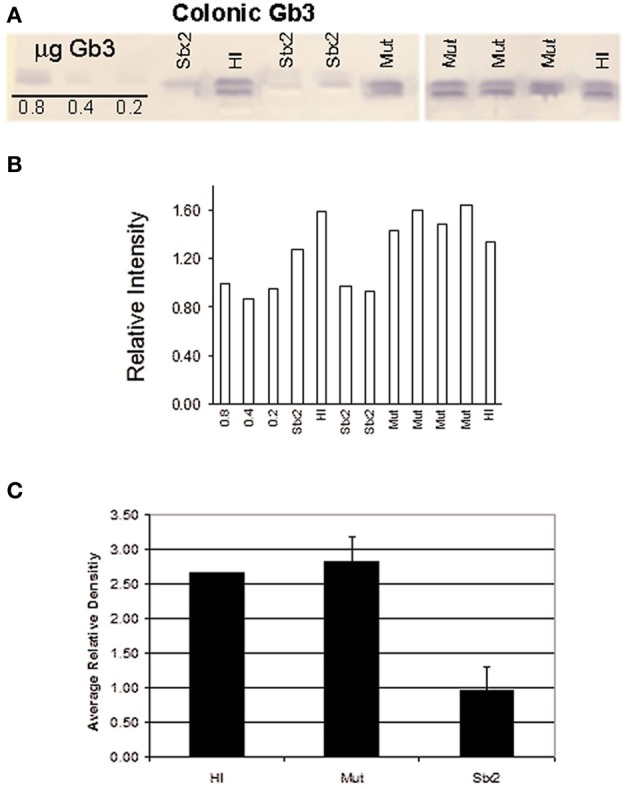
**(A)** Thin layer chromatography of extracted lipids from the distal colon tissue of 2 HI, 4 Mut-, and 3 Stx2-treated infant rabbits. Gb_3_ was detected using a Shiga toxin 1 B-subunit overlay method (Nutikka et al., 2003). Standards of 0.8, 0.4, and 0.2 μg Gb3 are shown on the far left of the TLC plate. **(B)** TLC band density for each sample was determined using ImageJ 1.38× software (Rasband, 1997–2007). **(C)** Mean density values were plotted for each treatment group. Error bars represent the standard deviation.

**Figure 6:**
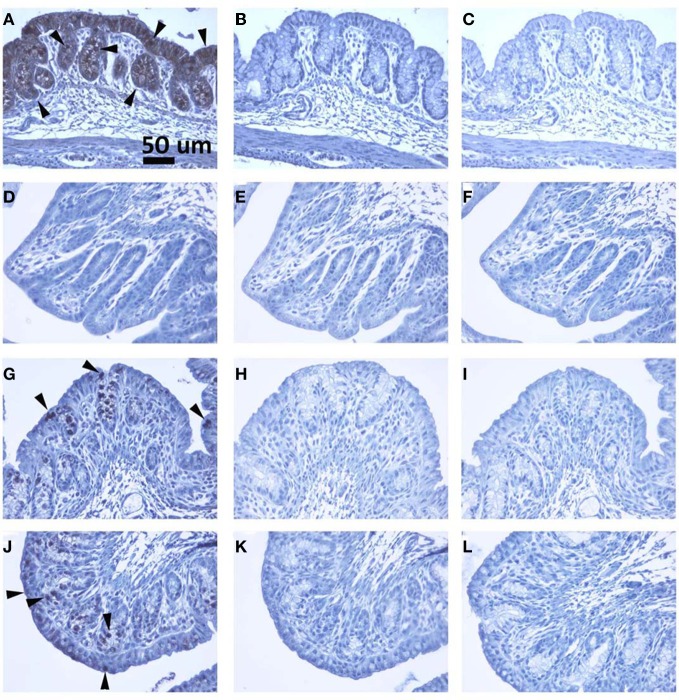
**(A–C), (D–F), (G–I)** and **(J–L)** are colon sections from naïve, Stx2, heat-inactivated Stx2 and Stx2 mutant treated rabbits, respectively. **(A, D, G** and **J)** are sections stained with anti-Gb_3_ antibody. Strong signal in A (luminal layer) and less intense stain in G and J can be recognized, whereas no signal is detected in D (Stx2 treated). Arrowheads show examples of Gb_3_ positive spots. In **(B, E, H** and **K)**, isotype matched rat IgM was used in the place of anti-Gb_3_ antibody to serve as a negative control. **(C, F, I** and **L)** are primary antibody omitted, buffer control (negative control). All controls shown are negative. A scale bar in **(A)** indicates 50 mm.

Recent studies suggest that the human colonic epithelium expresses Gb_3_ (Zumbrun et al., 2010). In rabbits Gb_3_ is maturationally regulated in the small bowel (Mobassaleh et al., 1994), which is why wide cell intestinal apoptosis is observed in adult rabbits challenged with Stx in ileal loop models (Keenan et al., 1986). In contrast, nothing is known about Gb_3_ levels in the rabbit colon. To determine if Gb_3_ is present in colonic tissue of 5-day old rabbits, 1 cm tissue sections were subjected to lipid extraction followed by thin-layer chromatography. Gb_3_ was detected in the distal colon of animals challenged with HI or Mut, while Stx2-treated animals appeared to have little or no Gb_3_ (Figures 5 and 6). Similarly, immunohistochemical staining of tissue sections from infant rabbit colon revealed the presence of Gb_3_ (Figure 6), which decreases following Stx treatment. Therefore, infant rabbit colon expresses Gb_3_.

### Stx2 induces phosphorylation of p38 in infant rabbit colon

**Figure 7:**
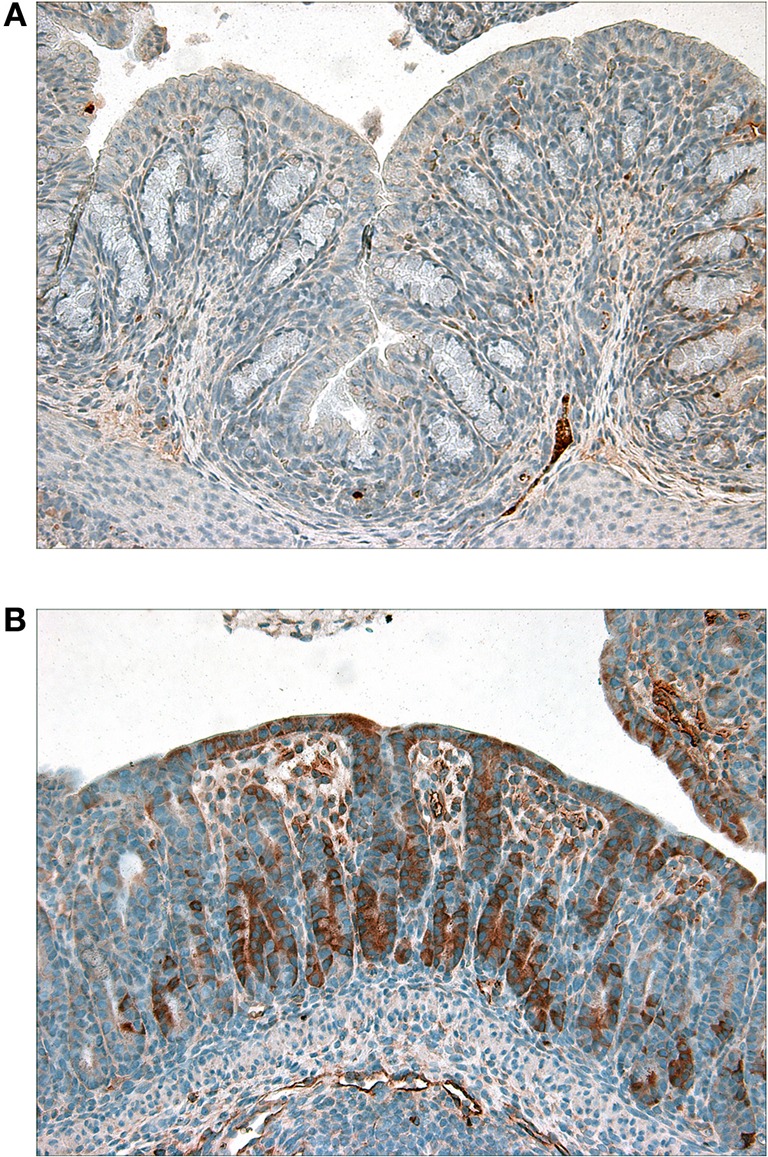
**Immunohistochemical staining of phosphorylated-p38 in colon sections from (A) Mut- or (B) Stx2-treated infant rabbits.** Dark brown staining is indicative of the presence of phosphorylated p38.

In previous studies, we have shown that Stxs induce the RSR in the human colonic epithelial cell line HCT-8 (Smith et al., 2003), and that the resultant activation of the MAPKinases p38 and the Jun N-terminal kinases (JNKs) are essential for induction of several proinflammatory cytokines. We therefore investigated whether p38 activation could be detected in the colon of Stx2-treated animals (Figure 7). Although present in both samples, intestinal levels of P-p38 are higher in Stx2-treated compared with Mut-treated infant rabbits suggesting that p38 is indeed activated in response to Stx2.

We have also shown that inhibition of p38 diminishes Stx2-induced pro-inflammatory cytokine production in intestinal epithelial cells (Thorpe et al., 1999a). To determine whether inhibition of p38 activity *in vivo* results in decreased colonic inflammation, we pre-treated infant rabbits by intraperitoneal injection with the p38 inhibitor SB203580. Following intoxication with Stx2, no significant histopathological differences were detected between SB203580-treated vs vehicle treated animals (data not shown).

### Effects of the ZAK inhibitor DHP-2 on Stx2-induced colonic inflammation

We next chose to target the MAPKinase pathway more proximally at the MAP3Kinase level. The MAP3Kinase ZAK has been shown to induce the Stx-mediated RSR *in vitro* (Jandhyala et al., 2008), and we have demonstrated that the ZAK specific inhibitor DHP-2 (Wang et al., 2005) was effective at the inhibition of Stx2-induced activation of p38 and JNKs in HCT-8 cells. Therefore, we investigated whether DHP-2 could prevent Stx2-induced pathology. Infant rabbits were treated with DHP-2 by oral gavage prior to Stx2 intoxication. As with the SB203580 treatments (data not shown), no significant difference in histopathology was detected between vehicle and DHP-2 treated rabbits.

### Treatment with the tyrosine kinase and ZAK inhibitor imatinib reduces heterophil infiltration in response to Stx2

Ricin cleaves the same adenine from the α-sarcin/ricin loop of the 28S rRNA as Stx and initiates a similar ribotoxic stress response, resulting in increased pro-inflammatory gene expression (Iordanov et al., 1997; Smith et al., 2003). In 2010, Lindauer et al. demonstrated that nilotinib and sorafenib could block ricin mediated p38 and JNKs activation (Lindauer et al., 2010). Nilotinib and sorafenib are drugs used to treat myelogenous leukemia and renal cancer respectively by inhibiting the tyrosine kinase activities of the oncoprotein BCR-Abl (nilotinib) or of vascular endothelial growth factor receptor and platelet-derived growth factor receptor (sorafenib). However, studies have also shown that these and other antineoplastic drugs, including the BCR-Abl inhibitors dasatinib and imatinib, have affinity to and/or can inhibit additional kinases including ZAK (Karaman et al., 2008; Manley et al., 2010). Because these kinase inhibitors are known to be pharmacologically active *in vivo*, have been well-studied in mouse models, and are approved for use in humans, we thought they might be good candidates for testing in our infant rabbit model.

**Figure 8:**
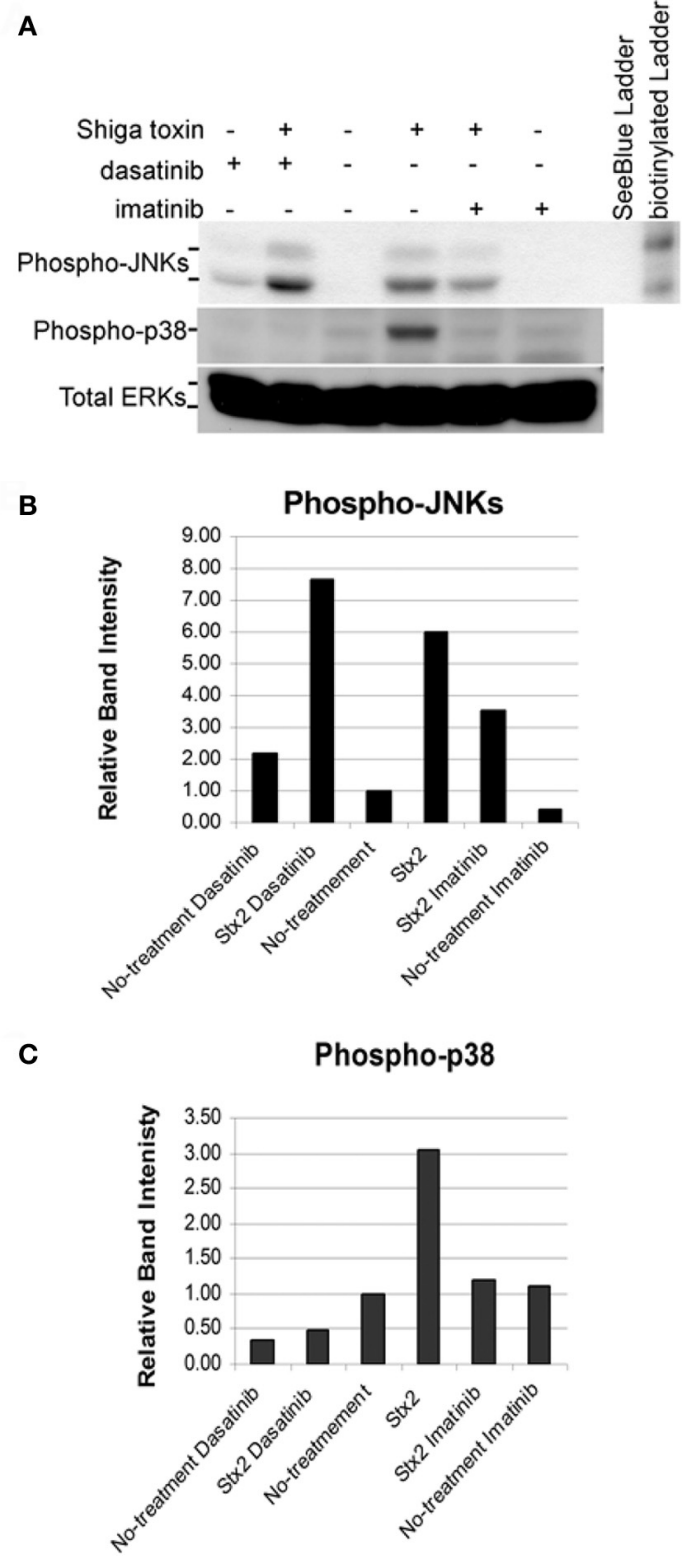
**Confluent HCT-8 cells were pretreated for 1 h with DMSO (vehicle), 200 μM imatinib, or 200 μM dasatinib.** Cells were then left untreated or treated with 5 μg/ml of Stx2 for 4 h. 150 μg of protein from each whole cell lysate was loaded in each lane. **(A)** Western blots were performed for phospho-JNKs, phospho-p38, and total ERKs (loading controls). Graphical representation of normalized band intensities are shown for phospho-JNKs **(B)** and phospho-p38 **(C)**.

We tested the effects of imatinib and dasatinib on inhibition of Stx2-induced ribotoxic stress *in vitro* using HCT-8 cells. Both imatinib and dasatinib when administered at 200 μM had a clear inhibitory effect on Stx2-induced p38 phosphorylation (Figures 8A and C). However, only imatinib showed inhibition of Stx2-induced JNKs phosphorylation (Figures 8A and B) while dasatinib at 200 μM appeared to promote JNKs phosphorylation even in the absence of Stx2. Despite imatinib having a relatively low affinity for ZAK, (*K*_*d*_ = 2.6 μM) compared to dasatinib (*K*_*d*_ = 45 nM), nilotinib (*K*_*d*_ = 8–10 nM), and sorafinib (*K*_*d*_= 6.3 nM) (Karaman et al., 2008; Manley et al., 2010) we chose to test imatinib in our rabbit model as it was readily available at our hospital pharmacy in the form of a pharmaceutical-grade tablet.

**Figure 9:**
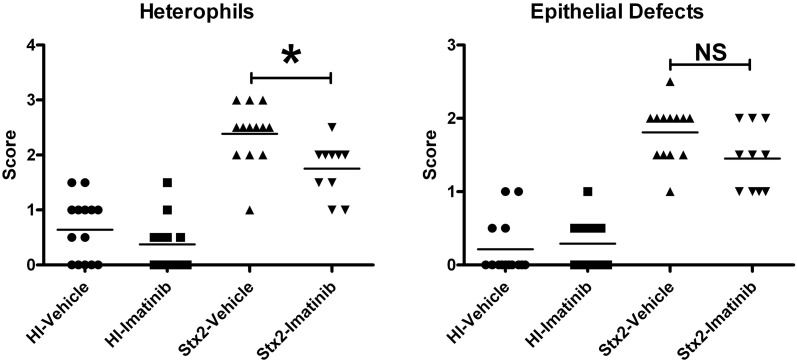
**Animals were treated with 125 mg/kg imatinib 2 times daily beginning 12 h prior to HI or Stx2 dosing.** The data represents blinded scoring for heterophils and epithelial defects. ^*^Significant *P*-value = 0.0068; **NS** (not significant) *P*-value = 0.064. HI-vehicle *n* = 14, HI-imatinib *n* = 12, Stx2-vehicle *n* = 13, and Stx2-imatinib *n* = 10.

We elected to administer imatinib to infant rabbits using a dose of 125 mg/kg every 12 h by oral gavage. Of the 49 rabbits used for this study, 23 were treated with Stx2 (13 with vehicle and 10 with imatinib), and 26 were treated with HI (14 with vehicle and 12 with imatinib). All Stx2-treated animals developed diarrhea regardless of inhibitor or vehicle pre-treatments. However, the imatinib-treated animals (Figure 9) had statistically less heterophil infiltration into the intestinal mucosa (*P* = 0.008) compared with those animals pre-treated with vehicle, suggesting that the imatinib did confer some protective effect. There was also a slightly lower mean score (non-significant *P* = 0.06) for epithelial defects in the imatinib treated animals. At the dose we used, there was no effect of imatinib administration on basal inflammation observed in control (HI-treated) animals.

## Discussion

One of the barriers to the development of therapeutics to treat Shiga toxin-mediated illnesses has been the lack of good small-animal models, in which oral challenge with STEC results in diarrhea followed by hemolytic uremic syndrome with renal and neurological pathology. Small-animal models for studying STEC generally fail to consistently replicate all facets of STEC-mediated disease (Proulx et al., 2001). For example, although the infant rabbit has proven to be very useful for studying intestinal colonization by STEC strains (Ritchie et al., 2003, 2011; Ritchie and Waldor, 2005; Ho et al., 2008; Vazquez-Juarez et al., 2008), the animals do not develop thrombotic microangiopathy. Because STEC-mediated disease clearly starts in the intestine, and the role of Stxs in intestinal disease is poorly understood, the infant rabbit model may be a useful one in which to study how Stxs can contribute to intestinal damage during STEC infection. The widely accepted assertion that the Stx receptor Gb_3_ is absent in the human intestinal epithelium, confines Stx-mediated effects to the Gb_3_-positive intestinal microvasculature, myofibroblasts, neuroendocrine cells, and Paneth cells and has resulted in downplaying the role of Stxs in intestinal pathology (Schuller et al., 2004, 2007; Schuller, 2011). However, by demonstrating that Stxs do bind to human colonic epithelium, and that Gb_3_ synthase, the enzyme that synthesizes Gb_3_ from lactose ceramide, is expressed in these cells, a study by Zumbrun et al., 2010 was able to conclude that Gb_3_ is expressed in the human intestinal epithelium albeit at low levels (Zumbrun et al., 2010). Therefore Stxs may play a more significant role in promoting intestinal damage during STEC infection than some have perceived.

A large obstacle to understanding the role of Stxs in STEC-mediated intestinal disease is that STEC strains differ with respect to the arsenal of virulence factors they express, thereby making it difficult for researchers to reach a consensus as to which virulence factors are necessary or most important for promoting intestinal pathology and (presumably) subsequent systemic uptake of Stxs. Several virulence factors, including flagellins and factors associated with the locus of enterocyte effacement (LEE) have been implicated as some of the more important contributors to intestinal pathology during STEC colonization (Ritchie et al., 2003; Rogers et al., 2003; Miyamoto et al., 2006). Therefore, in order to fully evaluate the role of Stxs in intestinal disease, the use of models in which toxins can be effectively administered in the absence of other known virulence determinants is important.

In this study, we use the infant rabbit as a model to investigate specifically Stx-induced intestinal pathology. We show that Stx2-induced colonic inflammation in infant rabbits is associated with A-subunit activity, cannot be induced by holotoxin in the absence of *N*-glycosidase activity, and is associated with activation of the SAPKinase p38. These findings are all consistent with the hypothesis that Stx2 induces the RSR in this model. Unlike the small bowel of rabbits in which Gb_3_ is maturationally regulated and toxin binding of Gb_3_ in jejunal microvillus membranes can only be detected after day 16 of life (Mobassaleh et al., 1988), our studies demonstrate that Gb_3_ is present in the colon by day 5. While it has been suggested that for cytotoxicity, endocytosis, and trafficking through the Golgi apparatus, Stx requires cell surface Gb_3_ localized in lipid rafts (Falguieres et al., 2001; Utskarpen et al., 2010), other studies have concluded that Stxs are able to enter cells independent of Gb_3_ (Malyukova et al., 2009). However, the extent of cellular damage caused by Stx following Gb_3_-independent entry was not determined in those studies. It should be noted that Gb3-independent macropinocytosis of Stx as proposed by Malyokova et al., may still contribute to intestinal inflammation by exposing Gb3-positive intestinal endothelial, myofibroblast, and neuroendocrine cells to Stx. Also, Stx2 but not Stx1 treatment of human intestinal biopsy cultures revealed epithelial cytotoxicity with pronounced cell extrusion from the crypt rims and crypt centers (Schuller et al., 2004). Interestingly, immunofluorescent staining of this tissue for Stx2 revealed strong staining of the lamina propria in proximity to pericryptal myofibroblasts, scattered stromal cells, and the epithelium. Together these data support a role for Stx2 in promoting damage to the human intestine during STEC infection.

Our observations that Gb_3_ is not detectable following Stx2 treatment supports the hypothesis that Stx2 is entering cells via this receptor and that intoxicated cells are either dying or are prevented from regenerating new Gb_3_. That the same magnitude of decrease does not occur in either HI- or Mut-treated animals suggests the enzymatic activity of the wild type Stx2 is promoting the loss of detectable Gb_3_ in these tissues. At the same time, the decrease in epithelial cell Gb_3_ in both HI-treated and Mut-treated animals compared to untreated animals is interesting, and may be worthy of further study. It is possible that the Stx2 catalytic mutant is bound to and taken up by Gb_3_-positive cells resulting in a decrease of detectable Gb3 at the surface of the cells, and that Gb_3_ is then replaced or regenerated as intoxication does not occur. It is also possible that similar events occur with our heat-inactivated preparation of Stx2 because it may retain B-subunit activity, as holotoxin, in general, is very heat stable (unpublished observations, Jandhyala et al.). Indeed, Fischer et al. have demonstrated *in vitro* that treatment of A498 cells with StxB but not holotoxin results in the release of free ceramide, suggesting that interaction of Gb_3_ with StxB may promote hydrolysis of the glycosphingolipid (Fischer et al., 2007).

Based on these data, we suggest that the infant rabbit is a useful model to study the contribution of Stx2 to various aspects of disease pathogenesis during the diarrheal phase of human illness. It may also be a useful model in which to study the exact mechanism(s) by which Shiga toxins breach the epithelial barrier in order to be taken up from the intestinal compartment into the systemic circulation. However, one obvious limitation to our study is that we did not assess our animals for the presence or absence of an alternate Stx receptor such as globotetraosylceramide (Gb_4_), nor did we specifically evaluate the role for Gb_3_ engagement by Stx2 on the host inflammatory response or apoptosis. These are areas that may be of interest for future studies.

Our attempts to use the infant rabbit model to test potential therapeutics directed at inhibiting the Stx-mediated MAPKinase response had mixed results. There are several important inherent difficulties in studying potential therapeutics in this specific model that are worthy of mention. First, little or nothing is known about appropriate dosing of the candidate therapeutics in the infant rabbit. When possible, we used doses and routes of administration of therapeutic candidates that had been successful in other studies, and/or estimated dosing intervals based on the assumption that infant rabbit metabolism would be similar to the metabolism of adult mice. Infant rabbits are slightly larger than adult mice, usually between 50 and 100 g on day 0, and they grow rapidly during the 48 h of the study. However, it is purely speculative as to how this observation may reflect the metabolism of a given compound. Second, infant rabbits typically feed once daily, but may feed more often, thus their stomach contents can vary greatly at different times of the day. Also, not all animals in a litter may feed equally well. Therefore, absorption of orally administered compounds may differ between individuals. Finally, enteric flora is being established in the first week of life, and may therefore be highly variable between litters and even within litters, which may certainly affect immunologic responses in the intestine.

Pre-treating the rabbits with the p38 specific inhibitor SB203580 resulted in no observed effect compared with vehicle treated controls. We chose a dose that was comparable to that used in murine models of arthritis and endotoxin shock (Badger et al., 1996), but it may have been insufficient for our model. Alternatively, blocking the other MAPKinase pathways downstream of ZAK such as JNKs and the extracellular receptor kinases (ERKs), may be necessary in order to have a significant effect on colonic inflammation, as the power of this study was only strong enough to detect a difference greater than 1.00–1.5 scoring units. Similarly, negative results were obtained using the ZAK inhibitor DHP-2. It is possible that the lack of positive results are due to sub-optimal dosing as no data has been published with regard to the pharmacodynamics of this compound. The single published use of this compound only described the treatment of mice with DHP-2 by oral gavage 1 h prior sacrificing and dissecting the animals (Wang et al., 2005).

Previous studies of EHEC and Stx using the infant rabbit model demonstrated that Stx contributes to EHEC-associated intestinal pathology by increasing heterophil infiltration (Ritchie et al., 2003). Interestingly, pre-treatment with imatinib which has been shown to have affinity for ZAK, seemed to have a modest, but statistically significant decrease of Stx2-induced heterophil infiltration. This is of particular note since it has been proposed that infiltration of polymorphonuclear leukocytes (PMNs) may promote systemic uptake of Stxs (Hurley et al., 2001). Indeed, treatment of the intestinal cell line HCT-8 results in the induction of the potent neutrophil chemo-attractant interleukin-8 (IL-8) (Thorpe et al., 1999b), and there is a correlation between numbers of PMNs and increased IL-8 in both patients with HUS and rabbits treated with Stxs (Fitzpatrick et al., 1992; Garcia et al., 2008). Our finding that imatinib can block Stx2-induced RSR events in intestinal epithelial cells *in vitro*, and decreases heterophil infiltration *in vivo*, supports our hypothesis that Stx2-induced ZAK activation is an upstream event contributing to the Stx2-induced colonic inflammation observed *in vivo*. However, future experiments aimed at evaluating MAPKinase activation and downstream gene activation will be needed to confirm that these observations are due to ZAK inhibition versus the inhibition of one or more other kinases. Also, future efforts to evaluate the therapeutic potential of this class of agents will focus on other drugs in this family, such as nilotinib, which has a much higher affinity for ZAK (~8–10 nM) than imatinib (2.6 μM) (Karaman et al., 2008; Manley et al., 2010) and has been shown *in vitro* to block the RSR (Sauter et al., 2010). We have also recently developed a *zak* knockout murine line with a targeted deletion that will be useful to further assess the role of ZAK activation in ribotoxic stressor-induced inflammatory events *in vivo*.

In conclusion, we have demonstrated that Stx2A-subunit activity is necessary for intestinal pathology, including apoptosis, in the colon of infant rabbits. We have also demonstrated that a cell surface receptor for Stx, Gb_3_, is present in the infant rabbit colon and provided data suggesting that Stx2 engages these Gb_3_-positive cells. Finally, we have provided for the first time, *in vivo* data showing that a compound with affinity for ZAK, may lessen Stx2-induced intestinal damage. Together these data support the infant rabbit model as a reasonable small-animal model for studying Stx-mediated intestinal disease and evaluating candidate therapeutics for treating Stx-mediated intestinal inflammation.

### Conflict of interest statement

The authors declare that the research was conducted in the absence of any commercial or financial relationships that could be construed as a potential conflict of interest.
